# Exploration of Preliminary Objective Triage by Menopause Score and CA 125 Result Prior to Accelerating Fast-Track Booking for Suspected Ovarian Cancer—A Role for the Pathway Navigator?

**DOI:** 10.3390/diagnostics14050541

**Published:** 2024-03-04

**Authors:** Robert Woolas, Lisa Young, Dirk Brinkmann, Francis Gardner, Richard Hadwin, Thomas Woolas, Natalia Povolotskaya

**Affiliations:** 1 Department of Gynaecological Oncology, Portsmouth Hospitals University Trust, Portsmouth PO6 3LY, UK; dirk.brinkmann@porthosp.nhs.uk (D.B.); francis.gardner@porthosp.nhs.uk (F.G.); natalia.povolotskaya@porthosp.nhs.uk (N.P.); 2 Wessex Cancer Alliance, Southampton SO16 4GX, UK; 3 Southampton University Hospitals Trust, Southampton SO16 6YD, UK; 4 Department of Mathematics & Science, University College London, London WC1E 6BT, UK; tom.woolas.17@alumni.ucl.ac.uk

**Keywords:** risk of malignancy index, ovarian cancer diagnosis triage, Serum CA 125, pathway navigator

## Abstract

The 28-days-to-diagnosis pathway is the current expected standard of care for women with symptoms of ovarian cancer in the UK. However, the anticipated conversion rate of symptoms to cancer is only 3%, and use of the pathway is increasing. A rapid triage at the moment of receipt of the referral might allow resources to be allocated more appropriately. In secondary care, multidisciplinary teams (MDTs) use the risk of malignancy index (RMI) score, (multiply menopausal status pre = 1 or post = 3 × ultrasound score = 0 − 3 × the CA 125 level), using a score of >200, to triage urgency and management in possible ovarian cancer cases. The most powerful determinant of the RMI score variables is CA 125 level, an objective number. Could a simple modification of the RMI score retain a high sensitivity for cancer whilst improving specificity and, consequently, decrease the morbidity of false-positive classification? To test this hypothesis, a retrospective evaluation of an ovarian two-week-wait telephone clinic of one consultant gynaecological oncologist was undertaken. Enquiry re menopause status was scored as one for pre- and three for postmenopausal or uncertain. CA 125 levels of >67 u/mL for premenopausal and >23 u/mL for postmenopausal women were used to precipitate urgent cross-sectional imaging requests and MDT opinions. These CA 125 cut thresholds were calculated using an assumption that the RMI imaging score, regardless of whether the result was available, could be three. We contemplate that women who did not exceed a provisional RMI score of >200 might be informed they are extremely unlikely to have cancer, removed from the malignancy tracker and appropriate follow-up arranged. One hundred and forty consecutive cases were analysed; 43% were deemed premenopausal and 57% postmenopausal. Twenty of the women had cancer, eighteen (90%) of whom had an RMI > 200. One hundred and twenty were benign, and only twenty-three (19%) classified as urgent cases in need of accelerated referral to imaging. In contrast, CA 125 > 35 u/mL, whilst retaining the sensitivity of 90%, misclassified 36 (30%) of the benign cases. It is possible that a telephone triage via a questionnaire determining menopausal status and the CA 125 result could offer a sensitivity for cancer of 90% and urgent expert review of under 20% of benign cases. This rapid initial telephone assessment could be presented by a trained pathway navigator, physician associate or nurse specialist. Substantial savings in NHS cancer services resources, anxieties all around and reduced patient morbidity may occur as a result.

## 1. Introduction

NHS England has mandated a 28-day faster diagnosis standard for all cancer patients [1]. Ovarian cancer is recognised to be a disease that is often characterised by a non-specific multitude of disparate symptoms and frequently leads to recriminations of missed diagnostic opportunities during previous health care consultations [2]. The National Institute of health & Care Excellence (NICE) have reviewed such symptoms and provided guidance on referral criteria to fast-track pathways [3]. However, they have also acknowledged that the specificity of this approach will be poor [4]. In addition, given existing postpandemic challenges to both primary and secondary care, there is an escalating risk of diagnostic systems being overloaded and potentially distracted from the 5–10% of pathway entrants who are seriously ill [5].

In this context, it is relevant to explore the epidemiology of ovarian malignancy, as the performance characteristics of the available tests alter substantially after the menopause. Almost all (95%) of ovarian cancer is epithelial in origin [6]. The majority of these cases occur, through accumulation of mutational damage with time, in the usual, postmenopausal, cancer age group. Within this epithelial group, a distinct morphological subset of borderline or low malignant potential lesions can be defined, characterised clinically by presentation on average 10–20 years prior to their high-grade aggressive poor-prognosis counterparts, invariably at stage 1 and with consequent long-term survival rates of over 80% [7]. A plethora of pathologies constitutes the 5% of non-epithelial lesions which are more evenly distributed across the whole age spectrum. Unlike other site-specific referral recommendations, no lower age limits are set on the ovarian pathway guidance [3]. This may be because of the bimodal distribution of ovarian cancer presentation caused by aggressive but curable germ cell lesions presenting in young women, the 50% of borderline tumours that present in premenopausal years and the effect of familial cancer syndromes whose germline mutations manifest as early-age-onset cases. In total, approximately 15% of cases present prior to the age of 50 years [8].

Furthermore, research observations over the last 30 years have re-evaluated the likely true origin of high-grade serous “ovarian cancer”, the most common and lethal variant, as arising from the distal fallopian tube [8,9]. Consideration of these findings may influence the choice of appropriate diagnostic tests [10].

CA 125 is 40 years old [11]. Measurement of serum levels quickly became established as a useful discriminator of malignant from benign pelvic masses and in monitoring therapeutic response and relapse of established disease [12]. At the time of diagnosis, using the traditional cut-off level of >35 u/mL will identify 85% of ovarian cancers, but only around 50% in patients with stage 1 disease [11,13]. It is a somewhat non-specific ovarian tumour marker. False-positive elevations are often seen in inflammatory conditions affecting coelemic surfaces, pericardium, pleura and peritoneum, as well as disseminated malignancies of extraovarian origin. At any one time, up to 15% of premenopausal women will have a serum level of greater than 35 u/mL. Among postmenopausal women, the prevalence of malignant pathology is greater, and conditions such as endometriosis or pelvic inflammatory disease are rare, thus improving the specificity substantially [14]. Also, it has been shown among this older group that persistent elevated levels are associated with a 5-fold increased risk of mortality within 5 years [15]. With these aspects in mind, investigation of CA 125 as a screening tool for symptomatic women in primary care has been undertaken and yielded encouraging information [16]. The potential discriminatory value of an inexpensive blood test, where very high levels invariably indicate significant pathology, makes measurement of this serum antigen logical as a prior requirement of ovarian fast-track referral [3].

Ultrasound scan or on occasions CT and or MRI to visualise ovarian morphology is also a helpful diagnostic tool, and it is invariably appropriate to initiate such a test for suspected pelvic pathology. However, ultrasound or imaging is not mandated by NICE as essential to trigger fast-track referral for suspected ovarian cancer [3]. In contrast to a blood test, these investigations may be logistically complicated to arrange, are more time consuming and expensive, are equipment- and trained operator-dependent in their interpretation and also lack specificity, particularly in premenopausal dynamic and functional ovaries [17]. Although reproducible validated scoring systems exist for defining benign or malignant patterns, their interpretation requires clinical expertise and judgement [18].

Some 35 years ago, Jacobs et al. described a risk of malignancy-index scoring system among women presenting with an adnexal mass, having defined the relative importance of the above three parameters using logistic regression [19]. Menopausal status was scored as one and three, respectively, for pre- and postmenopausal women. A relatively simple listed interpretation of the ultrasound features described in the report offered a score of zero, one or three for increasing complexity and consequent likelihood of cancer. Then, the CA 125 value was incorporated into the multiplication of the three factors as the actual serum level, a number ranging from 3 u/mL to over 1000 on some occasions. 

Multiple validations of this system are present in the literature, with minor variations and or improvements sometimes proposed [20]. Sensitivities and specificities of between 80 and 90% can be achieved, with different thresholds trading one value off against the other. However, perhaps because it is reproducible and the simple mental calculation can be performed in a few seconds, today, RMI scores of 200 (more sensitive) or 250 (more specific) are written into protocols for objective classification of MDT management choices around the world.

The NHS faster diagnosis cancer publication [1] also describes a novel job role: that of the pathway navigator. This is envisaged as a non-clinical appointment, but fundamentally entails communicating directly with the patient and collating available clinical information, including ensuring that primary care-initiated diagnostic tests such as a CA 125 result is available as soon as possible. A brief questionnaire including enquiry about menopausal status could also be completed via the initial telephone contact.

In the interest of expedient management, we wish to explore the hypothesis that multiplying the CA 125 value by menopausal status of one or three could offer an immediate triage to very high risk, intermediate or low risk before the imaging is available, interpreted or even requested. This might allow the high-risk and potentially sick patients to be instantly highlighted to all concerned and the low-risk patients to be reassured that although they had entered a cancer diagnostic pathway, it is extremely unlikely that this diagnosis will prove to be the case. This could be achieved within a few hours of receipt of the referral email that commences the pathway in secondary care.

## 2. Materials and Methods

To test the potential of this approach, the dataset of a solo consultant ovarian faster diagnosis first-contact telephone clinic was retrospectively updated with final clinico/pathological outcomes and subsequently investigated. All initial telephone contacts were conducted by one individual who had asked women about their menopausal status, looked for a recent CA 125 result and enquired whether recent imaging and investigations had been performed or an appointment was pending. In the absence of available or imminent results, urgent investigation was initiated. Individual cases were managed according to clinical judgement. Age, menopausal status, symptoms, past history, any relevant family history, drug therapies and co morbidities were recorded, and the RMI was calculated as soon as data from investigations were available. A score of 200 or greater is the threshold used by our MDT to centralise surgery for care of women referred with a pelvic mass, and the same threshold was set in this retrospective study to imply arrange urgent cross-sectional imaging, face-to-face discussion and MDT review.

Because imaging information was not always available at the time of first contact, but the CA 125 result was more frequently known, the concept of a provisional RMI score (pRMI) was conceived as follows. 

The cut-off thresholds of CA 125 described in the results were chosen as follows and are illustrated in Table 1.

Using the original RMI methodology of multiplying M × U × CA 125 = ? > 200

A menopause score of 1 or 3 was ascribed following discussion with the patient. 

Regardless of whether a scan result was available, it was assumed that all women had a U score of 3. (A worst-case scenario). 

The implication of this imaging assumption was that, given that the premenopausal multiple was 1 × 3 = 3, any CA 125 above 67 u/mL would become a provisional RMI score > 200.

The postmenopausal multiplication was by 3 × 3 = 9; therefore, the critical CA 125 number to achieve >200 score was 23 u/mL.

Calculating the RMI when only 2 of the 3 variables were known with certainty was described as the provisional version (pRMI).

## 3. Results

We analysed 140 consecutive patients with definite outcome data available in our hospital system.

All patients received a telephone call between 1 and 14 days following electronic receipt of the referral by the secondary care institution. The variability of this time span reflected both the date and time of referral and availability of an appropriate appointment slot. 

CA 125 or imaging test results available at the time of first phone contact with the patient are illustrated in Table 2.

The ages ranged from 18 to 88 years. Of the analysed patients, 43% were deemed premenopausal and 57%—postmenopausal. In only two (1.5%) cases was it impossible to discern this status with certainty. Missing CA 125 data were invariably corrected within 72 h and the imaging was updated or arranged over a more variable time period.

A total of 41 (29%) of 140 patients had a pRMI score of >200. Eighteen had cancer and twenty-three had benign pathology. The positive predictive value of cancer in those who tested positive (pRMI > 200) was 44%.

Table 3 illustrates the significance of menopausal status in discriminating these two pRMI > 200 groups. In the premenopausal group, the chance of cancer was one in five, and after the menopause, over one in two women had malignant disease. Benign pathologies (false positives, but not conditions without consequence), as expected included endometriosis, congestive cardiac failure, diverticular disease, sarcoidosis, liver cirrhosis, and pelvic inflammatory disease.

The 20 patients diagnosed with cancer are listed in Table 4. Fourteen women had primary ovarian cancer and six had malignant diseases arising from other sites. All six (100%) of these women were correctly identified as pRMI > 200, consequently fast-tracked through our service and, following MDT opinion, were referred directly to the appropriate site-specific team meeting. We perceive this rapid transit as advantageous to these women. Furthermore, among this group, it is possible that the pelvic ultrasound will be normal, as independent pathology present, for example, in the chest, may be the cause of the symptoms and raised CA 125 level. 

Ninety-seven women were correctly identified as likely benign through a pRMI of <200. Two women with cancer, both postmenopausal, were also categorised as benign by the pRMI scores of 56 and 108 respectively. 

## 4. Discussion

Consideration of recruitment bias:

This clinic was set up to accommodate fast-track access for women who were referred with possible ovarian cancer. Although not the specified intention, it is possible that selected higher-risk cases were given appointments for this service in particular, as it offered direct access into the gynaecological oncology team.

Evidence supporting this possible source of bias could be the predominantly postmenopausal patients and perhaps the high risk of having cancer diagnosed (14%).

Clarification of this contention was not possible, as a gynaecological organ of suspected disease was not differentiated in our institution’s fast-track data capture system.

This concern is relevant, as if the prevalence of benign cases increased, although the sensitivity would remain accurate, the positive predictive value recorded would probably diminish.

The origin of the CA 125 cut-off levels (Table 1):

Conventionally, CA 125 has a cut off-level of >35 u/mL as abnormal. Indeed, this is the level acknowledged by NICE and primary care as needing referral for further investigation. It has been suggested that a different level of normal should be applied to premenopausal women [21], but this has never been included in laboratory reports. The RMI, by noting the occurrence menopause or not, incorporates this physiological age difference into its calculation. In the pRMI > 200 described above, the two levels defining triage of 67 and 23 u/mL arise because the convenient number of 200 has been chosen (although this number was defined as near optimum based on the original receiver operator characteristic curve). These levels have not been discussed in the literature before, but their juxtaposition around the conventional 35 u/mL level illustrates the principle underlying our hypothesis. It is necessary to increase the sensitivity in the postmenopausal group, where malignant pathology is most prevalent, and increase the specificity in the premenopausal women, in whom false positives are more likely. This should enable us to focus our resources more appropriately. However, determining to what extent this action compromises sensitivity overall becomes the ultimate challenging question of our proposal. 

An alternative RMI score of 250 has also been considered by some authors [14]. In our pRMI calculation, the two numbers would be 84 and 28 u/mL, respectively. In the small series presented here, there is little difference compared to the results obtained by using this threshold. 

Test result availability (Table 2). 

The data presented in Table 2 appear somewhat disconcerting, as only 35% of patients referred had both diagnostic tests easily available at the time of the appointment. Numerous explanations and reasons could be discussed, which include too quick a response by secondary care, as acquisition of data may take time. However, the most redeemable would appear to be that although the fast-track referral form may be triggered by a scan report accessible to the primary care team, this may not be possible to see via our hospital system. In addition, perhaps because there is no mandated requirement for an ultrasound scan in NG 12, there is no clear guidance indicating that requesting one might save patients from waiting longer for an opinion. Furthermore, the blood result component of the referral proforma is separate from the ovarian component, potentially leading to omissions. Also, access to phlebotomy is often somewhat patient-dependent, especially in the elderly, as they may be requested to make a convenient appointment themselves.

Age and malignant pathology: Influence on sensitivity.

Table 3 illustrates that advancing age as a predictor of likely malignant pathology should always be regarded as associated with higher risk. These observations have been documented before, as has the propensity for elevated levels of CA 125, undertaken as a primary care test for symptomatic women, to antedate the diagnosis of other sites of malignant disease as well as ovarian cancer [22]. In our study, over 25% of the malignancies were non-gynaecological cancers. All six of these women were correctly identified as pRMI > 200, were consequently fast-tracked through our service and, following MDT opinion, were referred directly to the appropriate site-specific team meeting. We perceive this as advantageous to these women and would certainly not describe these cases as false-positive individuals. In addition, there is a propensity for these cases to present as advanced malignancy, where decisions not to aggressively pursue precise questions about the origin of the tissue may be made. Furthermore, even detailed studies of outcomes relating to diagnosis and mortality of ovarian cancer may be left with uncertainty about the most likely choice of primary site that the available information can offer. Thus, some inaccuracy in classification of primary site of origin of cancer is inevitable in any dataset [23].

Therefore, we are content to analyse these data as malignant disease vs. no malignancy, but are aware that precise data on ovarian cancer also need to be taken into account and will certainly be needed if examining cancer registry and treatment outcomes information.

The two false-negative results (pRMI < 200):

Both of the false negatives occurred among the primary ovarian cancers. One patient had no histology but had ovarian cancer recorded as her cause of death. She was 86 and had a large mass that had been known about for some years, but intervention had been declined by the patient, who had many co morbidities. We believe this opportunity to intervene earlier might have been quite successful, as clearly the malignant potential of the tumour was not especially aggressive when first detected and radiological assessment at 35 cms identified it as a granulosa cell tumour. Unfortunately, the patient died whilst being investigated with regard to fitness for surgery.

The second case was a 56-year-old postmenopausal woman with a 12 cm diameter stage 1 borderline mucinous cystadenocarcinoma. The mucinous variant of ovarian cancer is recognised to be the most likely to be associated with low serum levels of CA 125, which has raised interesting questions about the true origin of the widely metastatic variants, which may in fact be primary gastrointestinal tumours [24]. With regard to the borderline lesions, they are almost always still confined to the ovary at presentation; grow quite large, which testifies to the low grade, slow evolution and low metastatic potential of the lesion; may be associated with transitions from benign cystadenoma to borderline appearance through invasive cancer; and are invariably cured by surgery alone [25]. Although this is an epithelial ovarian cancer, the 10-year survival rate of stage I cases is in the order of 99%.

It is not our intention to delay such cases from receiving treatment. An ultrasound scan should be performed quickly and any pelvic mass needs to be evaluated and discussed face to face with a clinician. Invariably, surgery will be recommended (and hopefully expedited), but the precise date will inevitably depend on the symptoms and local departmental service circumstances at that time.

Hypothetical future false-negatives:

This modification to the pathway potentially predominantly misclassifies the younger premenopausal patients. Amongst this group, the CA 125 threshold has been raised by nearly 100% Therefore, we might anticipate a possible reduction in sensitivity for cancer. Population-based up-to-date genomic stratified data on who contracts ovarian cancer before the age of 50 are not available. However, early-age-onset cancer raises the question of genetic predisposition and evaluation. In particular, BRCA germline mutation testing will be offered as PARP inhibitor drug treatment, as it has consequent therapeutic relevance. Most cases of ovarian cancer have now been offered testing in the UK for almost a decade. Approximately 15% of index case women with a non-mucinous tumour have a mutation, and the mean age of presentation of a BRCA1-positive woman is 40 years. HGSC tumours predominate [8] and, as shown in Table 4, these are the lesions that generally express CA 125 in abundance. Only a substantially larger dataset will be able to show whether this effect offsets the higher threshold of CA 125 in premenopausal women needed to triage into our high-risk group. 

Regardless, we believe that knowledge of the relevance of a cancer family history is spreading rapidly amongst our population, and increasing numbers of people from at-risk families may be able to self-identify this during their initial primary care consultation. It may be that a future version of an RMI score may be modified to enhance performance by incorporating additional genetic or family history scores. Meanwhile, in such cases where death of a first-degree relative may be graphically described, the increased perceptions of risk and consequent emotions expressed clearly come with an overlay of additional anxieties, and therefore fast-track data acquisition seems entirely appropriate. 

Comparison of pRMI score and utility of CA 125 alone >35 u/mL threshold: 

Table 5 presents the difference achieved by pRMI in contrast to using the CA 125 result alone at the 35 u/mL cut off. As predicted by our hypothesis the pRMI test has superior specificity and positive predictive value to CA 125 alone and apparently confers no loss of sensitivity. We acknowledge that the number of malignant events is small (*N* = 20) and especially so amongst premenopausal women (*N* = 2), both of whom had raised (>67 u/mL) CA 125 levels. However, the 1 to 9 pre and postmenopausal distribution is compatible with the ratio of pre to postmenopausal women affected by lethal ovarian cancer in the general population. Therefore, that neither of these premenopausal cases were adversely affected by raising the CA 125 threshold to 67 u/mL is encouraging and additional detailed study of a larger group of such patients is warranted.

**Figure 1:**
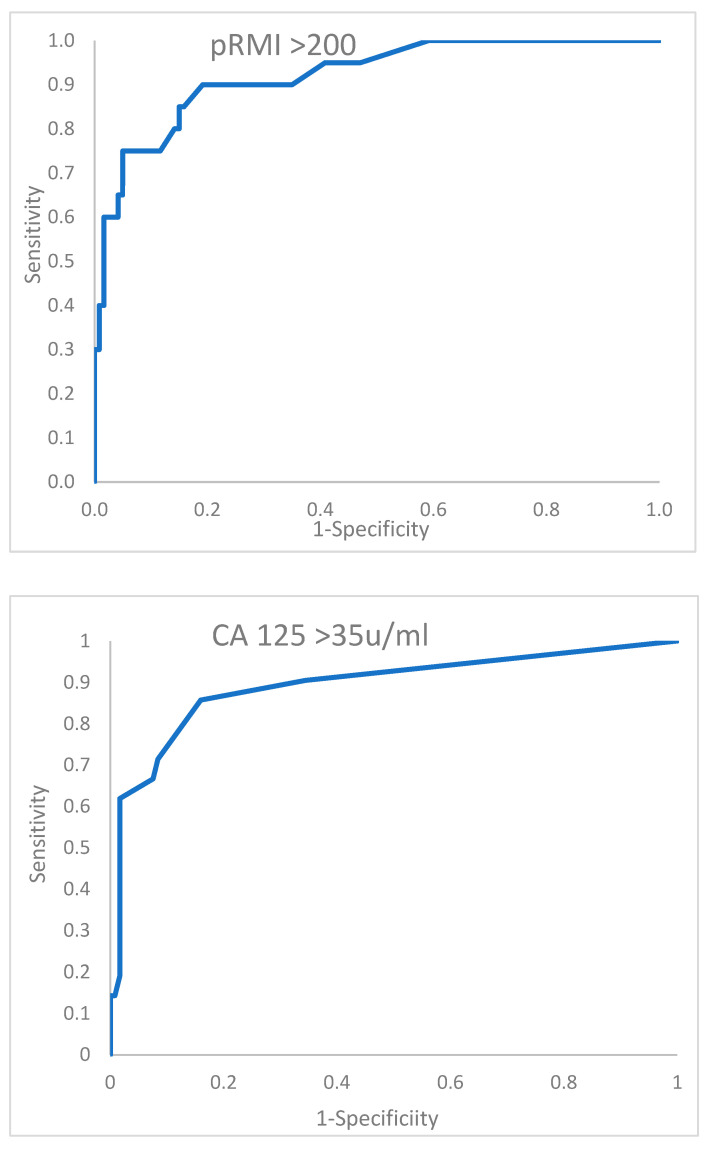
Receiver operator characteristic curves of provisional RMI (PRMI) compared to that of CA 125 > 35 u/mL alone.

The performance of the two tests above is further described by the receiver operator characteristic curves shown in Figure 1. At a glance, these curves do not appear dramatically different, and only an insignificant advantage in area under the curve is conferred by the pRMI test. We believe that the small size of the database containing only 20 malignant events contributes to this modest difference. In addition, only 13 benign cases of the 140 total subjects changed classification through application of the pRMI score. This represents an 11% shift in the benign allocation, which visually appears to be a limited influence. However, it should be considered that NICE have indicated that the criteria for urgent cancer referral should be set at a relatively low 3% conversion rate [3], whereas the ratio in this series of malignant to benign cases is 14.3%. Therefore, in national practice, possibly a 5-fold difference in the total number of benign cases would be observed, potentially influencing a much larger number of women, who could be instantly reassured they are very unlikely to have a malignant diagnosis.

Would the sensitivity for cancer be retained? Only a much larger dataset can adequately answer these questions. These data presented represent an underpowered study. As we only have 20 patients with malignant pathology, we currently have a power of 19.2%. Our estimate of the number of women in each group required to have the power to confirm our hypothesis with 90% certainty is that 180 women with malignant disease would be needed, implying analysis of a dataset of over 1000 patients.

Impact on patient outcome: 

It is important to appreciate that urgently scheduling every fast-track case will be unlikely to influence the ultimate physical prognosis of those that turn out to have ovarian cancer. A prospective NIHR, be clear on cancer study was performed in 2013 in the north-west of England [26]. A well-organised and -delivered local symptoms campaign reported an increase in GP contact and use of CA 125 assays, increased volume of ovarian fast-track referrals, but no additional ovarian cancers, no stage shift and, on longer follow up, no appreciable impact on survival from ovarian cancer. In addition, unfortunately, both the United States and the twenty-year duration UK randomised controlled trials of screening for ovarian cancer, amongst even earlier-phase asymptomatic postmenopausal women, were unable to detect a survival benefit to screening healthy individuals with either ultrasound or serum CA 125 assay [27,28].

We therefore conclude that the important likely tangible benefit of a fast-track pathway is relief of patients’ psychological distress through speedy, succinct communication that someone empathic to their case is initiating rapid clarification of uncertainty. An objective clinical opinion of likely outcome should be explained, especially as the majority of patients entering the pathway are ultimately not going to receive a diagnosis of cancer. Also, whilst awaiting diagnosis, symptomatic management (and hopefully alleviation of physical discomfort) can be achieved.

In addition, quickly identifying and prioritising cases in which a rapidly evolving pathophysiological process leads to patients deteriorating, perhaps beyond the point where they could tolerate treatment with either surgery or chemotherapy at all, is important, as a delay could reduce their life expectancy substantially.

This latter situation is extremely pertinent to ovarian cancer, in which the most lethal variant of the disease, high-grade serous carcinoma (HGSC), has a propensity to spread intraperitoneally with associated disrupted mesothelial cells, leading to membranes porous to protein and fluids with consequent intraperitoneal ascites and pleural fluid formation in conjunction with rapid deterioration of a self-perpetuating hypoproteinaemic and intravascular volume-depleted state. In a matter of a few days, especially among elderly patients with other comorbidities, the individual may pass beyond the window of opportunity in which one could reasonably safely suggest an operation and/or primary chemotherapy [29].

In addition, it should be considered that these high-grade, aggressive, rapidly evolving lesions are also found in the patients who might expect a good initial response and, consequently, achieve remission if given the chance to receive platinum-based chemotherapy.

Interestingly, Table 4 also illustrates that these cases are the ones most likely to express high serum levels of CA 125. Our triage system is therefore predisposed to identify these cases most easily and with almost 100% sensitivity. We believe that amongst these women, who are all likely to end up with a diagnosis of serious pathology, the speed of service response will make the most physical difference to their welfare, especially if treatment is delivered before irreversible decompensation occurs, potentially increasing an individual’s life expectancy by a number of years.

## 5. Conclusions

Among 140 women urgently referred with suspected ovarian cancer, phone contact, acquisition of knowledge of menopausal status and current serum CA 125 level could identify 90% of the cancer among 20% of the referrals.

These numerical data could be used to inform how best to allocate available urgent appointment slots in a resource-constrained service.

Furthermore, the remaining 80% of the urgent referrals could be quickly verbally reassured that they are very unlikely to receive a diagnosis of cancer.

We remain concerned by some loss of sensitivity for malignancy (?10%). However, clinical interpretation of available imaging information as it becomes available could obviously override this triage process.

The increased susceptibility of younger women to false-negative diagnoses might be offset by asking about family history of cancer and requesting germ cell tumour-marker assays.

## Figures and Tables

**Table 1:** Logic of precise CA 125 cut thresholds for provisional RMI (pRMI) calculation.

Menopausal Status	M	U Predict	CA 125 u/mL	pRMI	Risk Stratification
Premenopausal	1	3	<66	<198	Low/intermediate
Premenopausal	1	3	>67	>201	High risk
Postmenopausal	3	3	<22	<198	Low/intermediate
Postmenopausal	3	3	>23	>207	High risk

U predict = ultrasound-assumed score which, in provisional RMI calculation, is 3.

**Table 2:** Test result electronic availability at time of first patient appointment.

Result Availability ^1^	Scan not Performed/Unknown	Scan Result Available
CA 125 not performed/unknown	40 (29%)	7 (5%)
CA 125 result available	43 (31%)	50 (35%)

^1^ These data, as discussed later, do not represent an accurate reflection of referral practice.

**Table 3:** Women with pRMI > 200 by menopausal status and benign or malignant diagnosis.

p RMI > 200 *n* = 41	Premenopausal *n* = 10	Postmenopausal *n* = 31
Benign *n* = 23	8	15
Malignant *n* = 18	2	16

**Table 4:** Details of all 20 cases of cancer.

Age	Meno Statu	CA125 u/mL	ProvRMI	Primary Site	Diagnosis
60	ost	7	56	B EOC	Stage Ia borderline mucinous ovarian cancer in 16 cm multilocular unilateral ovarian mass
69	post	13	108	? OC	No histology: 35 cm ovarian mass; slow-growing stage 1b on imaging? Granulosa cell: patient died
35	pre	73	219	Non EOC	Stage Ia squamous cell carcinoma in 12 cm ovarian dermoid cyst: post op pet scan negative
86	post	91	273	Pancreas	Pancreatic cancer bloated; no ovarian mass seen
88	post	36	324	Colon	Dukes’ B Colon cancer; obstructing lesion resected
77	post	60	540	Lung	Lung cancer; weight loss; no ovarian mass
58	post	73	657	EC + ? EOC	Bleeding + mass; endometrioid endometrial carcinoma grade 3 + bilateral ovarian masses involved
45	pre	258	774	Breast	Breast (BC) metastatic, prior history of early BC (BRCA -ve))
81	post	114	1026	EOC	HGSC stage III NACT + IDS
78	post	158	1422	EOC	HGSC stage III NACT + IDS
80	post	169	1521	Non-EOC	Neuro endocrine tumour in ovarian teratoma
51	post	186	1674	Lymphoma	Hodgkin’s lymphoma stage IV; chest wall involved
54	post	225	2025	EOC	HGSC/PPC stage III; surgery; no ovarian tumour
70	post	358	3222	EOC	HGSC/PPC stage IV; no ovarian mass seen; bilateral pleural effusions; 6 cycles of chemotherapy; no IDS
76	post	418	3762	EOC	HGSC stage IV; large ovarian mass; NACT; no IDS
68	post	543	4887	EOC	HGSC stage III; both ovaries enlarged
54	post	1011	9099	EOC	HGSC stage III BRCA1 mutation
65	post	2940	26,460	EOC	HGSC IV Bilateral 9 and 5 cm masses
79	post	3787	34,083	EOC	HGSC IV NACT IDS pending
86	post	4147	37,323	? OC	? Cancer: MDT imaging diagnosis stage IV; ovaries involved but primary anywhere; palliative care

EOC = epithelial ovarian cancer. B EOC = borderline epithelial ovarian cancer. Non-EOC = ovarian primary other than epithelial origin. EC = endometrial cancer. HGSC/PPC = High-grade serous carcinoma/primary peritoneal carcinoma (normal-sized ovaries). NACT = Neoadjuvant chemotherapy given as primary treatment rather than surgery. IDS = interval debulking surgery. Prov RMI = provisional risk of malignancy index calculation. Illustrated in Table 5 is a comparison of the novel pRMI score and an analysis using CA 125 alone at the conventional cut threshold of >35 u/mL. The performance of the two tests is further described by the receiver operator characteristic curves shown in Figure 1.

**Table 5:** Test performance CA 125 alone >35 u/mL vs. PRMI > 200 calculation.

Test	Sensitivity	Specificity	Positive Predictive Value
CA 125 alone >35 U/mL	90%	70%	33%
PRMI value > 200	90%	81%	44%

## Data Availability

Not applicable.

## References

[1] https://www.england.nhs.uk/cancer/faster-diagnosis/.

[2] Dilley J., Burnell M., Gentry-Maharaj A., Ryan A., Neophytou C., Apostolidou S., Karpinskyj C., Kalsi J., Mould T., Woolas R. (2020). Ovarian Cancer Symptoms, Routes to Diagnosis and Survival—Population Cohort Study in the “no Screen” Arm of the UK Collaborative Trial of Ovarian Cancer Screening (UKCTOCS). Gynecol. Oncol..

[3] www.nice.org.uk/guidance/NG12.

[4] (2011). The Recognition and Initial Management of Ovarian Cancer.

[5] Razai M.S.M., Majeed A. (2022). General Practice in England: The Current Crisis, Opportunities, and Challenges. J. Ambul. Care Manag..

[6] Russell P., Farnsworth A. (1997). Surgical Pathology of the Ovaries.

[7] McCluggage W.G. (2010). The Pathology of and Controversial Aspects of Ovarian Borderline Tumours. Curr. Opin. Oncol..

[8] Berek J.S., Renz M., Kehoe S., Kumar L., Friedlander M. (2021). Cancer of the Ovary, Fallopian Tube, and Peritoneum: 2021 Update. Int. J. Gynaecol. Obstet. Off. Organ Int. Fed. Gynaecol. Obstet..

[9] Woolas R., Jacobs I., Davies A.P., Leake J., Brown C., Grudzinskas J., Oram D. (1994). What Is the True Incidence of Primary Fallopian Tube Carcinoma?. Int. J. Gynecol. Cancer.

[10] Woolas R.P., Oram D.H., Brown C.L., Jacobs I.J. (1997). Primary Carcinoma of the Pelvic Peritoneum Intercepted by Screening for Ovarian Cancer. Int. J. Gynecol. Cancer.

[11] Bast R.C., Klug T.L., St. John E., Jenison E., Niloff J.M., Lazarus H., Berkowitz R.S., Leavitt T., Griffiths C.T., Parker L. (1983). A Radioimmunoassay Using a Monoclonal Antibody to Monitor the Course of Epithelial Ovarian Cancer. N. Engl. J. Med..

[12] Jacobs I., Bast R.C. (1989). The CA 125 Tumour-Associated Antigen: A Review of the Literature. Hum. Reprod..

[13] Woolas R.P., Xu F.-J., Jacabs I.J., Yu Y.-H., Daly L., Brechuck A., Soper J.T., Clarke-Pearson D.L., Oram D.H., Bast R.C. (1993). Elevation of Multiple Serum Markers in Patients with Stage I Ovarian Cancer. J. Natl. Cancer Inst..

[14] Woolas R.P., Jacobs I.J., Shepherd J.H., oram D.H., Blackett A.D., Luesley D.M., Berchuck A., Hudson C.N. (2003). Serum tumour markers in the clinical management of ovarian cancer. Ovarian Cancer.

[15] Jeyarajah A.R., Ind T.E., MacDonald N., Skates S., Oram D.H., Jacobs I.J. (1999). Increased Mortality in Postmenopausal Women with Serum CA125 Elevation. Gynecol. Oncol..

[16] Hamilton W., Round A., Sharp D. (2009). Ovarian Cancer. Not a Silent Killer. BMJ Clin. Res. Ed..

[17] Woolas R., Xu F.J., Daly L., Soper J.T., Berchuck A., Rodriguez G., Clarkepearson D., Boyer C.M., Bast R.C. (1993). Screening Strategies for Ovarian Cancer. Diagn Oncol..

[18] Timmerman D., Planchamp F., Bourne T., Landolfo C., du Bois A., Chiva L., Cibula D., Concin N., Fischerova D., Froyman W. (2021). ESGO/ISUOG/IOTA/ESGE Consensus Statement on Pre-Operative Diagnosis of Ovarian Tumors. Int. J. Gynecol. Cancer Off. J. Int. Gynecol. Cancer Soc..

[19] Jacobs I., Oram D., Fairbanks J., Turner J., Frost C., Grudzinskas J.G. (1990). A Risk of Malignancy Index Incorporating CA 125, Ultrasound and Menopausal Status for the Accurate Preoperative Diagnosis of Ovarian Cancer. Br. J. Obstet. Gynaecol..

[20] Davies A.P., Jacobs I., Woolas R., Fish A., Oram D. (1993). The Adnexal Mass: Benign or Malignant? Evaluation of a Risk of Malignancy Index. Br. J. Obstet. Gynaecol..

[21] Malkasian G.D., Knapp R.C., Lavin P.T., Zurawski V.R., Podratz K.C., Stanhope C.R., Mortel R., Berek J.S., Bast R.C., Ritts R.E. (1988). Preoperative Evaluation of Serum CA 125 Levels in Premenopausal and Postmenopausal Patients with Pelvic Masses. Discrimination of Benign from Malignant Disease. Am. J. Obstet. Gynecol..

[22] Funston G., Hamilton W., Abel G., Crosbie E.J., Rous B., Walter F.M. (2020). The Diagnostic Performance of CA125 for the Detection of Ovarian and Non-Ovarian Cancer in Primary Care: A Population-Based Cohort Study. PLoS Med..

[23] Kalsi J.K., Ryan A., Gentry-Maharaj A., Margolin-Crump D., Singh N., Burnell M., Benjamin E., Apostolidou S., Habib M., Massingham S. (2021). Completeness and Accuracy of National Cancer and Death Registration for Outcome Ascertainment in Trials—An Ovarian Cancer Exemplar. Trials.

[24] Dundr P., Singh N., Nožičková B., Němejcová K., Bártů M., Stružinská I. (2021). Primary Mucinous Ovarian Tumors vs. Ovarian Metastases from Gastrointestinal Tract, Pancreas and Biliary Tree: A Review of Current Problematics. Diagn. Pathol..

[25] Gershenson D.M. (2017). Management of Borderline Ovarian Tumours. Best Pract. Research. Clin. Obstet. Gynaecol..

[26] Lai J., Mak V., Bright C.J., Lyratzopoulos G., Elliss-Brookes L., Gildea C. (2021). Reviewing the Impact of 11 National Be Clear on Cancer Public Awareness Campaigns, England, 2012 to 2016: A Synthesis of Published Evaluation Results. Int. J. Cancer.

[27] Buys S.S., Partridge E., Black A., Johnson C.C., Lamerato L., Isaacs C., Reding D.J., Greenlee R.T., Yokochi L.A., Kessel B. (2011). Effect of Screening on Ovarian Cancer Mortality: The Prostate, Lung, Colorectal and Ovarian (PLCO) Cancer Screening Randomized Controlled Trial. JAMA.

[28] Jacobs I.J., Menon U., Ryan A., Gentry-Maharaj A., Burnell M., Kalsi J.K., Amso N.N., Apostolidou S., Benjamin E., Cruickshank D. (2016). Ovarian Cancer Screening and Mortality in the UK Collaborative Trial of Ovarian Cancer Screening (UKCTOCS): A Randomised Controlled Trial. Lancet.

[29] Cavazzoni E., Bugiantella W., Graziosi L., Franceschini M.S., Donini A. (2013). Malignant Ascites: Pathophysiology and Treatment. Int. J. Clin. Oncol..

